# Genomic epidemiology of the clinically dominant clonal complex 1 in the *Listeria monocytogenes* population in the UK

**DOI:** 10.1099/mgen.0.001155

**Published:** 2024-01-02

**Authors:** Emily T. Fotopoulou, Claire Jenkins, Clare R. Barker, Anais Painset, Xavier Didelot, Ameze Simbo, Amy Douglas, Gauri Godbole, Frieda Jorgensen, Saheer Gharbia, Jim McLauchlin†

**Affiliations:** ^1^​ Water and Environmental Microbiology Services, UK Health Security Agency Food, 61 Colindale Avenue, London NW9 5EQ, UK; ^2^​ Gastrointestinal Bacteria Reference Unit, UK Health Security Agency, 61 Colindale Avenue, London NW9 5EQ, UK; ^3^​ Health Protection Research Unit in Gastrointestinal Infections, National Institute for Health and Care Research, University of Liverpool, Liverpool L69 7BE, UK; ^4^​ Health Protection Research Unit in Genomics and Enabling Data, National Institute for Health and Care Research, University of Warwick, Coventry CV4 7AL, UK; ^5^​ School of Life Sciences and Department of Statistics, University of Warwick, Coventry CV4 7AL, UK; ^6^​ Gastrointestinal Infections and Food Safety (One Health) Division, UK Health Security Agency, 61 Colindale Avenue, London NW9 5EQ, UK

**Keywords:** bioinformatics, data analysis, foodborne pathogens, *Listeria monocytogenes*, surveillance, whole-genome sequencing

## Abstract

*Listeria monocytogenes* is a food-borne pathogen, typically affecting the elderly, immunocompromised patients and pregnant women. The aim of this study was to determine the population structure of *L. monocytogenes* clonal complex 1 (CC1) in the UK and describe the genomic epidemiology of this clinically significant CC. We interrogated a working dataset of 4073 sequences of *L. monocytogenes* isolated between January 2015 and December 2020 from human clinical specimens, food and/or food-production environments. A minimum spanning tree was reconstructed to determine the population structure of *L. monocytogenes* in the UK. Subsequent analysis focused on *L. monocytogenes* CC1, as the cause of the highest proportion of invasive listeriosis in humans. Sequencing data was integrated with metadata on food and environmental isolates, and information from patient questionnaires, including age, sex and clinical outcomes. All isolates either belonged to lineage I (*n*=1299/4073, 32%) or lineage II (*n*=2774/4073, 68%), with clinical isolates from human cases more likely to belong to lineage I (*n*=546/928, 59%) and food isolates more likely to belong to lineage II (*n*=2352/3067, 77%). Of the four largest CCs, CC1 (*n*=237) had the highest proportion of isolates from human cases of disease (CC1 *n*=160/237, 67.5 %; CC121 *n*=13/843, 2 %; CC9 *n*=53/360, 15 %; CC2 *n*=69/339, 20%). Within CC1, most cases were female (*n*=95/160, 59%, *P*=0.01771) and the highest proportion of cases were in people >60 years old (39/95, 41%, *P*=1.314×10^−6^) with a high number of them aged 20–39 years old (*n*=35/95, 37%) most linked to pregnancy-related listeriosis (*n*=29/35, 83%). Most of the male cases were in men aged over 60 years old (40/65, 62%), and most of the fatal cases in both males and females were identified in this age group (42/55, 76%). Phylogenetic analysis revealed 23 5 SNP single linkage clusters comprising 80/237 (34 %) isolates with cluster sizes ranging from 2 to 19. Five 5 SNP clusters comprised isolates from human cases and an implicated food item. Expanding the analysis to 25 SNP single linkage clusters resolved an additional two clusters linking human cases to a potential food vehicle. Analysis of demographic and clinical outcome data identified CC1 as a clinically significant cause of invasive listeriosis in the elderly population and in women of child-bearing age. Phylogenetic analysis revealed the population structure of CC1 in the UK comprised small, sparsely populated genomic clusters. Only clusters containing isolates from an implicated food vehicle, or food processing or farming environments, were resolved, emphasizing the need for clinical, food and animal-health agencies to share sequencing data in real time, and the importance of a One Health approach to public-health surveillance of listeriosis.

## Abbreviations

CC, clonal complex; ESQ, Enhanced Surveillance Questionnaire; GBRU, Gastrointestinal Bacteria Reference Unit; LIPI-1, *Listeria* pathogenicity island 1; MLST, multilocus sequence typing; ST, sequence type; UKHSA, UK Health Security Agency; WGS, whole-genome sequencing.

## Impact Statement

By integrating genomic and epidemiological data linked to human cases of infection with *Listeria monocytogenes*, we showed that isolates belonging to CC1 caused the highest proportion of human clinical disease in the UK. We found that CC1 was commonly identified in females aged 20–39 with pregnancy-related listeriosis, and that mortality increased in patients over the age of 60 in both sexes. Phylogenetic analysis revealed the population structure of CC1 in the UK comprised small, sparsely populated genomic clusters exhibiting little evidence of being closely related in time and space. Contaminated food items associated with causing listeriosis in clinical cases were identified in clusters where the human isolate fell in the same 5 or 25 SNP single linkage cluster as the isolate from the implicated food vehicle, or food processing/farming environments. Contaminated food items causing clusters of human cases only were not identified based on epidemiological data (including food histories) extracted from the patient questionnaires. This finding emphasizes the need for clinical, food and animal-health agencies to share sequencing data in real time, and highlights the importance of a One Health approach to public-health surveillance of listeriosis.

## Data Summary

fastq files have been submitted to the National Center for Biotechnology Information (NCBI). All data can be found under BioProject no. PRJNA248549 and strain-specific information can be found in Table S1 (available with the online version of this article).

## Introduction

Listeriosis is a systemic infection caused by the Gram-positive bacterium *Listeria monocytogenes*. The genus *Listeria* comprises at least 26 species [1–4], and is ubiquitous in plant, water and soil environments [5]. *L. monocytogenes* and *Listeria ivanovii* are the two species within this genus known to cause infection in humans and animals, with *L. monocytogenes* being the leading cause of human clinical listeriosis [6]. After consuming foods contaminated with *L. monocytogenes*, patients may present with mild flu or gastroenteritis-like symptoms, although the causative agent is rarely isolated at the primary infection stage. Following the primary infection, patients over 60 years of age, the immunocompromised, pregnant women and their unborn or newly delivered infants, are at risk of developing listeriosis [7]. Infection in these high-risk groups can become invasive and manifest as bacteraemia, meningitis and at other extraintestinal sites [8]. Listeriosis infection during pregnancy may harm the unborn or newly delivered infant by causing miscarriage or still birth [9].

Human listeriosis is diagnosed through syndromic approaches in cases presenting with symptoms of invasive infection, followed by culture of the bacterium from a normally sterile site, most commonly from blood or cerebrospinal fluid [10]. The incubation period varies and has been reported to be between 1 and 91 days, and may correlate with severity [11–13]. In high-risk groups, listeriosis can be life-threatening; the Food and Drug Administration (FDA), World Health Organization (WHO) and UK Health Security Agency (UKHSA) report a mortality rate of 20–30 % [14–16]. Between 2010 and 2019, the UKHSA reported a mean case number for listeriosis of 160 cases per year in England and Wales [16].

*L. monocytogenes* can be classified into 13 different serovars based on flagella and surface antigens [17], with serovars 1/2a, 1/2b and 4b most commonly associated with human disease [18–20]. Based on phylogenetic analysis, *L. monocytogenes* is divided into four major lineage groups (I–IV), with I and II being the most prevalent [21]. The lineages are unequally distributed between clinical cases and food-production environments, with isolates belonging to lineage I more commonly recovered from human cases, while lineage II is more often associated with food and food-production environments [20]. Historically, different techniques have been used for typing *L. monocytogenes*, including PFGE, multilocus variable-number tandem-repeat analysis (MLVA), binary typing and PCR serotyping [22, 23]. However, for the most part, these have been superseded by whole-genome sequencing (WGS), which offers considerable advantages in terms of discrimination and comparability of data between laboratories [20].

WGS-derived multilocus sequence typing (MLST) offers accurate characterization of bacterial isolates based on the sequence variation (alleles) of a set of seven housekeeping genes [24]. Each allelic profile is assigned to a sequence type (ST), and similar STs are grouped together in clonal complexes (CCs) based on allelic variation [15]. A CC can, therefore, be defined as a collection of closely related STs, and this approach can be used to identify and define clonal groups within the population structure of *L. monocytogenes*. SNP typing methods offer an unprecedented level of strain discrimination, which can be used to provide information on source attribution and underpins surveillance of listeriosis by facilitating the detection and investigation of outbreaks [17].

As part of the UKHSA’s national surveillance remit, isolates of *L. monocytogenes* from all identified clinical human listeriosis cases are submitted to the Gastrointestinal Bacteria Reference Unit (GBRU) for WGS. In addition, the UKHSA Food, Water and Environmental (FW and E) Laboratory regularly collects samples from food and food-production environments to test for the presence of *Listeria* species, as part of routine surveillance, outbreak investigations and prevalence surveys. Since 2015, all *L. monocytogenes* isolates received by UKHSA have been characterized by WGS and sequences have been generated from >1000 clinical cases and >3000 food and food-production environment isolates. This has created one of the world’s largest longitudinal *L. monocytogenes* sequence databases.

Of particular interest are isolates of CC1, which belongs to lineage I and serovar 4b, with 20 % of all clinical isolates contained within the National Center for Biotechnology Information (NCBI) database are attributed to CC1 strains [25]. Worldwide population studies of *L. monocytogenes* have highlighted an abundancy of CC1, as well as its strong association with clinical cases [26–29]. CC1 clones have also been linked with various virulence factors, most commonly *Listeria* pathogenicity island 1 (LIPI-1) and *Listeria* pathogenicity island 3 (LIPI-3), and their significant association with severe clinical infection [30]. However, there is relatively little information of the distribution of CC1 clones causing human infection in England and Wales.

The aim of this study was to determine the population structure of *L. monocytogenes* CC1 in the UK and describe the genomic epidemiology of this clinically significant CC within the UKHSA database from January 2015 until December 2020.

## Methods

### Surveillance

The Health Protection (Notification) Regulations [31] require the operators of diagnostic laboratories to report the identification of *Listeria* species in human samples in the UK. Suspected cases are confirmed through isolation of the pathogen from clinical specimens from the infected site. Bacterial cultures are submitted to the Gastrointestinal Bacteria Reference Unit (GBRU) for species confirmation and sub-typing through WGS. Enhanced Surveillance Questionnaires (ESQs) are completed for all confirmed clinical cases, whenever possible. ESQs capture epidemiological information on clinical symptoms, food history, travel history and environmental exposures for 30 days prior to onset of illness. The national surveillance system for *Listeria* at UKHSA combines the epidemiological, microbiological and genomic data within a relational database containing laboratory samples collected from clinical cases and isolates from food, food components and food-production environments.

### Strain selection

There were 4073 unique isolates from food (*n*=2449), humans (*n*=928), environmental sources (*n*=618), undetermined sources (*n*=73) and animals (*n*=5) submitted to GBRU between 1st January 2015 and 31st December 2020 from Scotland, Wales, England and Northern Ireland in the GBRU archive. Of these, 237 *L*. *monocytogenes* CC1 isolates from food or environmental sources (*n*=77) and deduplicated human isolates (*n*=160) were selected for further study. Mother–baby pair isolates were considered as a single maternal–neonatal case. The temporal signal was assessed through the ‘receipt dates’ of the isolates, representing the date the isolates were received by GBRU from the diagnostic laboratories. Cases of listeriosis identified within 4 weeks of each other were defined as being temporally related. Cases residing within 100 km of each other were defined as being geographically related. Metadata such as sample source, sex, date of birth, postcode, patient type (revealing pregnancy-related cases) and disease outcome (recording patient deaths) were utilized for the purpose of this study. Due to wide food product circulation, geographical distribution and spatial signal was only assessed for clinical isolates through patient postcodes.

### WGS

Genome sequencing and sequencing analysis were carried out as previously described [20]. Bacterial isolates were sequenced using Illumina HiSeq 2500 and paired-end libraries were generated through the Nextera XT library preparation kit. Fragment size was assessed through the Perkin Elmer LabChip GX after fragmentation and clean-up. Paired-end fastq reads were trimmed using Trimmomatic, with bases below a phred score of ≤30 removed. Reads and pairs with a post-trimming length under 50 bp were discarded. To confirm the quality of sequencing, ensure no contamination and verify sample typing, an in-house *k*mer-based (a *k*mer is a short string of DNA with length *k*) approach was utilized (https://github.com/phe-bioinformatics/kmerid). Samples with non-*Listeria k*mers in the fastq reads were discarded.

The MLST ST (defined by the Pasteur scheme [32]) was extracted from each sequence using most (https://github.com/phe-bioinformatics/MOST) [33] and assigned a CC in accordance with the Institut Pasteur international MLST database for *L. monocytogenes* designation (http://bigsdb.pasteur.fr/listeria). Mapping was performed with reference strains selected based on CC identity. Isolates from CC1 were mapped to F2365 (GenBank accession no. AE017262). Burrows–Wheeler Aligner (BWA) Sequence Alignment Map outputs from were sorted and indexed, by SAMtools, to produce binary alignment maps [34]. A variant call format file was generated from each of the binary alignment maps by the Genome Analysis Toolkit 2 [35]. The variant call files were further parsed to extract only high-quality SNP positions. Pseudosequences of polymorphic positions were used to recreate maximum-likelihood trees by using iq-tree [36]. The fastq reads presented in this study can be found in the National Center for Biotechnology Information short-read archive under BioProject PRJNA248549.

### Phylogenies

To produce the maximum-likelihood phylogeny of the 237 CC1 isolates, a whole-genome alignment was first generated using SnapperDB (v0.2.8) [37], followed by Gubbins (v2.6) [38] to account for recombination-associated sequences. SnapperDB was then used once more, to generate an alignment of variants, where a given position was shared by a minimum of 80 % of the strains in the alignment and recombination regions were masked. iq-tree (v2.0.4) was used on this SNP alignment to produce a maximum-likelihood phylogenetic tree [via the general time reversible model (GTR) and 1000 bootstrap replicates]. Finally, tree visualization was achieved through the iTOL online platform (v5.7). Minimum spanning trees were reconstructed through GrapeTree (gpl v3) using the MSTree v2 algorithm and MLST profiles from the Pasteur Institute 7 loci scheme for *L. monocytogenes*. Hierarchical single linkage clustering (SLC) at the 250, 100, 50, 25, 10, 5 and 0 SNP level was used to define clusters of interest [37, 39–41]. Clusters of interest mapped on the phylogenies were derived by using hierarchical SLC at the 5 and 10 thresholds of pairwise SNP distances.

### Statistical analysis

Statistical analysis was performed through R [v 4.2.2 (2022-10-31)]. Fishers exact and chi-square tests were utilized to quantify the statistical significance of relationships between variables. Analyses with *P* values lower than 0.01 were considered as statistically significant.

## Results

### Population structure of *L. monocytogenes* in the UK

**Fig. 1.**
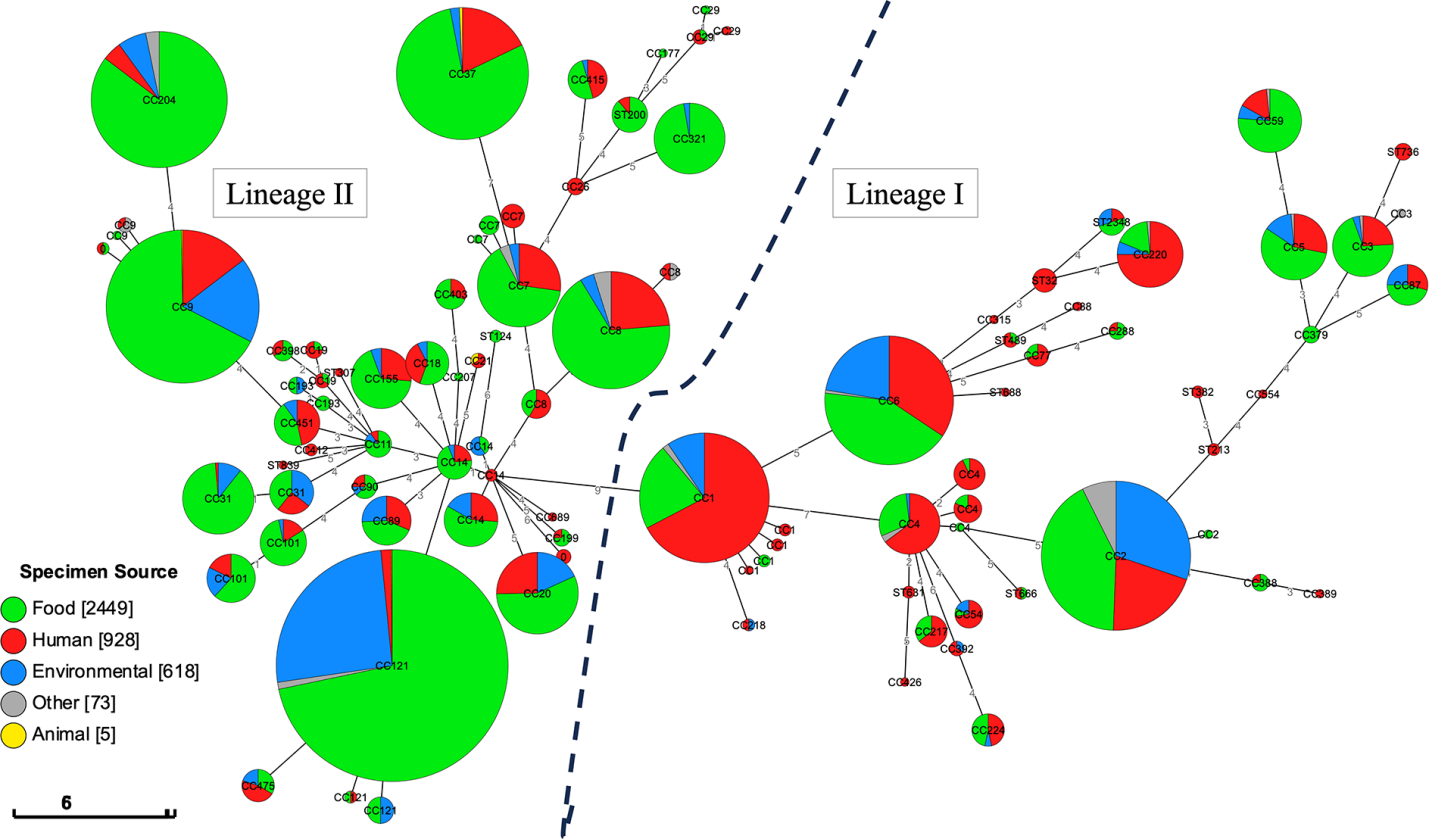
Minimum spanning tree showing the population structure. ST isolate clustering is shown with CC identity displayed on each cluster. Pie charts indicate cluster source disposition: isolates from food sources (green), isolates from humans (red), isolates from environmental sources (blue), isolates from undetermined sources (grey) and isolates from animals (yellow). A dotted line separates the isolates belonging to lineage II (left) from the isolates belonging to lineage I (right). The size of each pie-chart is indicative of the number of isolates belonging to each CC. The scale bar indicates expected changes per site per branch.

A minimum spanning phylogeny was reconstructed to represent the population structure of all *L. monocytogenes* isolates sampled and sequenced from England, Wales, Scotland and Northern Ireland between January 2015 and December 2020 (Fig. 1). All isolates belonged to either lineage I (*n*=1299/4073, 32%) or lineage II (*n*=2774/4073, 68%). CC source composition revealed an unequal distribution between clinical, food and/or food-production environments. Clinical isolates from human cases were more likely to belong to lineage I (*n*=546/928, 59 %, *P*<10^−5^) (Fig. 1, Table S1). In contrast, isolates sampled from food and/or food-production environments were more likely to belong to lineage II (*n*=2352/3067, 77%, *P*<10^−5^).

Analysis of the CC source composition enabled us to identify the clinically relevant CCs within the UK population structure of *L. monocytogenes*. Overall, the majority of isolates in this dataset belonged to CC121 (*n*=843), followed by CC9 (*n*=360) and CC2 (*n*=339) (Table S1). However, CC1 (*n*=237) had the highest proportion of isolates from human cases (*n*=160/237, 68%) compared to CC121 (*n*=13/843, 2%), CC9 (*n*=53/360, 15%) and CC2 (*n*=69/339, 20%) (*P*<10^−5^). Due to the enhanced clinical burden caused by CC1 in the UK, this CC was selected for further investigation.

### Descriptive epidemiology of CC1

**Fig. 2.**
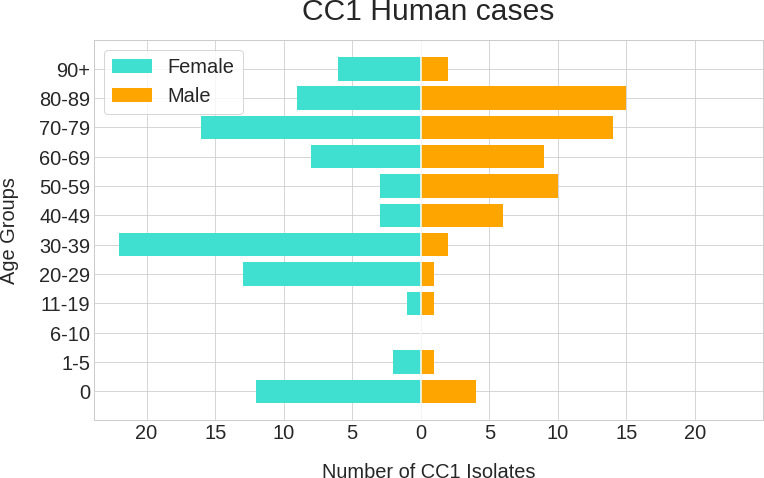
Human listeriosis case distribution across age (years) and sex for isolates belonging to CC1. A total of 160 isolates from human clinical cases from England, Scotland, Wales and Northern Ireland between 2015 and 2020 are shown in a comparative horizontal bar chart displaying listeriosis cases. Age is displayed in 10 (or 5) years group bins (*y*-axis) and the *x*-axis illustrates the number of cases. The blue bars illustrate the number of infections in females, in contrast to the orange bars male cases.

Listeriosis caused by isolates belonging to CC1 was most frequently associated with females (*n*=95/160, 59%, *P*=0.018) (Table 1). Most cases were vulnerable patients at the extremes of age, specifically infants aged <1 year old (*n*=16/160, 10%, *P*<10^−5^) and those aged >70 years old (*n*=62/160, 39%, *P*<10^−5^), or females aged 20 to 39 (*n*=35/160, 22%, *P*<10^−5^) (Table 1, Fig. 2). Of the total number of cases aged 20 to 39 years old, 35/38 (92 %) were female (Table 1, Fig. 2). A total of 83 % of female cases aged 20 to 39 were pregnancy linked (*n*=29/35), with 4 cases missing pregnancy association records. Notification of listeriosis were higher in female infants aged <1 year old (*n*=12/16, 75%, *P*=0.046); however, among the patients aged >70–89 years there was a significantly higher proportion of males (*n*=29/54, 53%, *P*=0.006) (Table 1, Fig. 2).

**Table 1.** Summary of age/sex and clinical outcome data for patients with listeriosis caused by *L. monocytogenes* belonging to CC1

	Sex and clinical outcome							
	Female	Male	Female		Male	Female	Male	Total
	**Survived**	**Survived**	**Pregnancy-related fatal case**	**Unknown**	**Unknown**	**Died**	**Died**	
**Age group**								
0	7	3	1	4	1	0	0	16
1 to 5	2	0	0	0	1	0	0	3
6 to 10	0	0	0	0	0	0	0	0
11 to 19	0	0	0	1	0	0	1	2
** **20–29	4	1	3	6	0	0	0	14
30–39	15	1	4	3	1	0	0	24
40–49	3	4	0	0	1	0	1	9
50–59	3	5	0	0	2	0	3	13
60–69	2	1	0	0	3	6	5	17
70–79	7	6	0	3	2	6	6	30
80–89	4	5	0	0	1	5	9	24
90+	1	1	0	1	0	4	1	8
Total	48	27	8	18	12	21	26	160

**Fig. 3.**
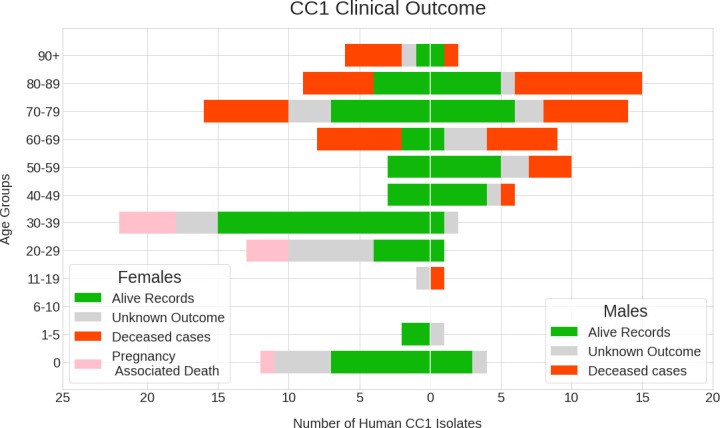
Age–sex pyramid including clinical outcome following infection with *L. monocytogenes* in each sex and all age groups (in years).

To assess clinical severity and the impact of listeriosis from CC1 clones, the clinical outcome of the infection was reviewed. In this dataset, although overall CC1 infection was more common in females, case fatality rate was significantly higher in males (males *n*=26/65, 40%, *P*=4.345×10^−5^; females *n*=29/95, 31%, *P*<10^−5^) (Table 1, Fig. 3). Fatal cases were most common in patients of both sexes aged >60 years (*n*=42/160, 26%). The proportion of fatal cases in females aged 60 and over was 54 % (*n*=21/39) compared to 14.3 % in those aged 60 and under (*n*=8/56) (*P*=0.005) (Table 1). The proportion of fatal cases in male patients 60 years old and over was 53 % (*n*=21/40), with a lower proportion in younger males (<60 years old) (*n*=5/25, 20%) (*P*=0.090).

To accurately capture all cases of pregnancy-related listeriosis, when no sample was collected from the mother, isolates sampled from neonatal cases were considered as pregnancy-related cases. There were 44/160 (27.5 %) infections caused by CC1 linked to pregnancy-related listeriosis, including miscarriage (pre-24 weeks pregnancy) and still births (post-24 weeks carried pregnancy) (Table 1).

### Temporal and spatial distribution

**Fig. 4.**
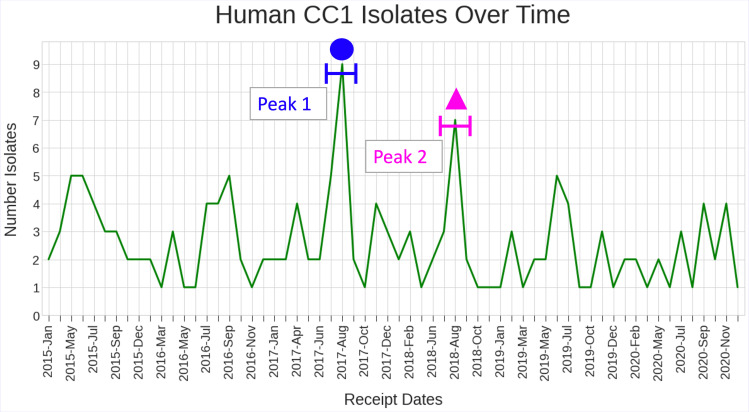
CC1 distribution of isolates sampled from hospitalized infected humans (*n*=160) from 2015 to December 2020.

**Fig. 5.**
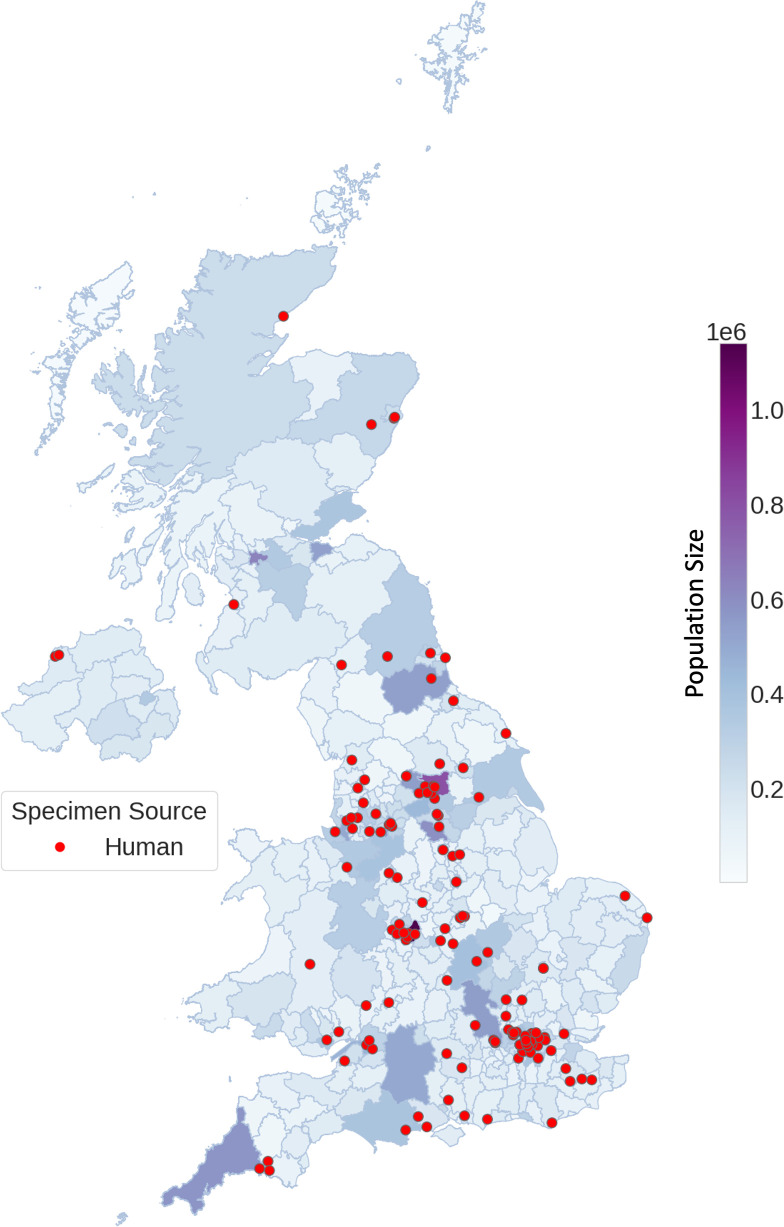
Spatial distribution map of CC1 isolates across England, Wales, Scotland and Northern Ireland. The map of the UK displays the UK's population density as a heat map, with the circle markers illustrating the geographical location of all clinical CC1 isolates. Population density is as described by the Office for National Statistics for 2019–2020.

From January 2015 to December 2020, the mean number of cases per month was 2.6 (Fig. 4). The case numbers per month ranged from one to nine cases, with most months not exceeding five cases per month, with the highest number of notifications occurring in August 2017 (*n*=9) and August 2018 (*n*=7) (Fig. 4). Cases were distributed across all regions of the UK (Fig. 5).

### CC1 phylogeny

#### Genomic analysis of phylogenetic clusters

**Fig. 6.**
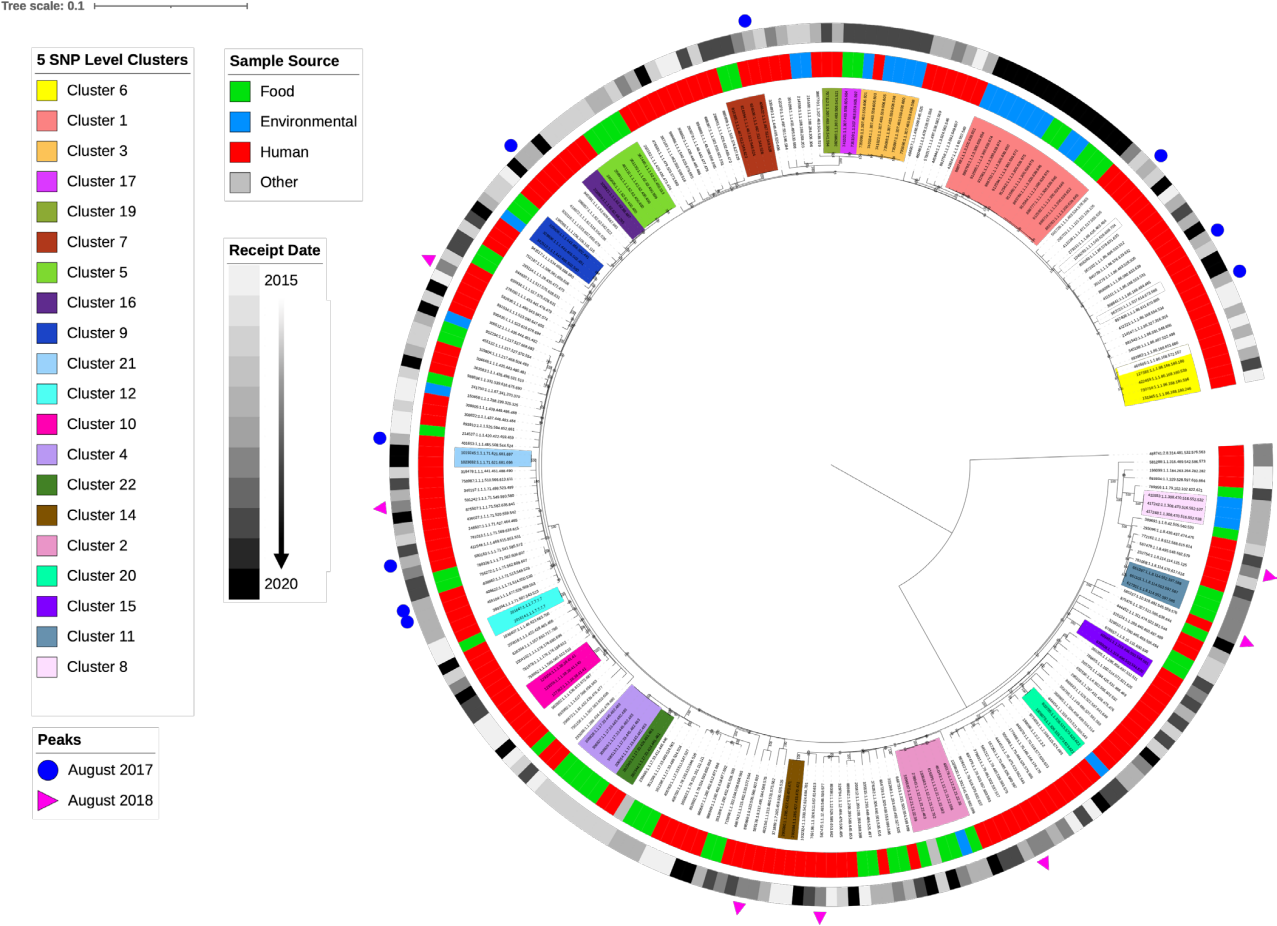
Phylogenetic analysis of isolates in CC1 (maximum-likelihood tree rooted on the most distant isolate). The first outer coloured strip ring illustrates the source of each isolate; human isolates (red), food isolates (green), environmental isolates (blue) and undetermined source of isolates (grey). The second colour strip ring is a sequential range of grey colours where the darkest colour represents the most recent date (December 2020), whereas the lightest colour indicates the oldest date (January 2015). The blue circles and pink triangles dispersed through the tree point to the isolates linked with two peaks in CC1 incidence during August 2017 and August 2018. The 20 highlighted clades on the tree represent clusters comprising at least two isolates within a ≤5 SNP difference. The scale bar represents number of mutations per site.

To examine the genetic diversity represented within the CC1 population in the UKHSA database, a phylogeny comprising whole-genome sequences from 237 isolates (human clinical cases *n*=160; food *n*=50; environment *n*=24; unknown source *n*=2) was generated (Fig. 6). Clusters were reviewed at the 5 and 25 SNP single linkage cluster level. Clusters comprising isolates that fell within 5 SNPs of another isolate from clinical human cases, food and environmental sources were linked to epidemiological data and listed in Table 2.

**Fig. 7.**
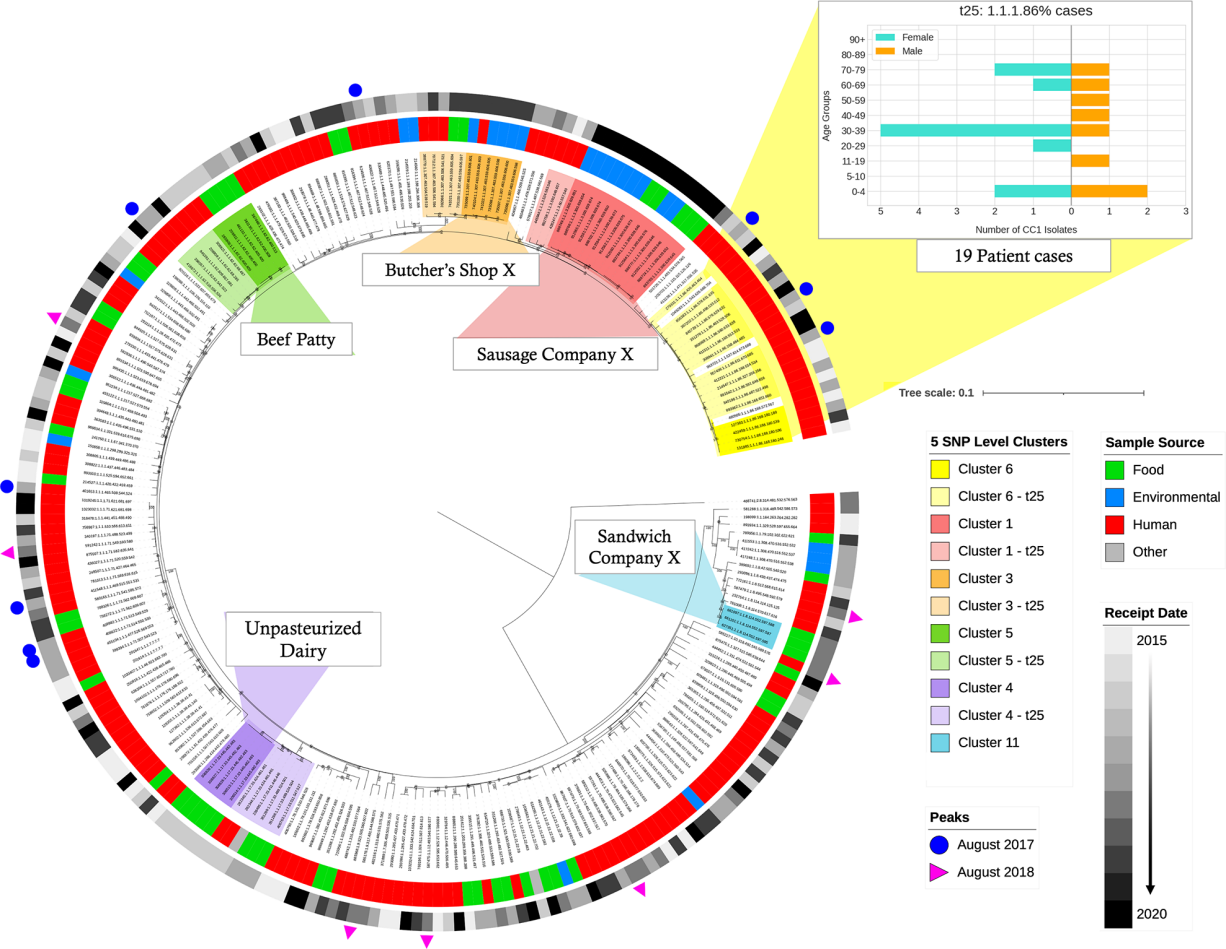
CC1 phylogeny with epidemiological clusters within a 25 SNP difference. Maximum-likelihood tree, rooted on the most distant isolate. The first outer colour strip ring illustrates the source of each isolate: human isolates (red), food isolates (green), environmental isolates (blue), and undetermined source of isolates (grey). The second colour strip ring is a sequential range of grey colours where the darkest colour represents the most recent date (December 2020), whereas the lightest colour indicates the oldest date (January 2015). The blue circles and pink triangles dispersed through the tree point to the isolates linked with two peaks in CC1 incidence during August 2017 and August 2018. The five highlighted clades on the tree represent clusters comprising at least five isolates within a 25 SNP difference. The darker colour clusters highlight the clusters at a 5 level, whereas the lighter colour clusters show isolates within 25 SNP difference. The bar chart linked to cluster 6 (yellow) graphically represents an age-sex pyramid of all human isolates within this cluster. The scale bar represents number of mutations per site.

**Table 2.** Clusters comprising of two or more isolates falling within a 5 SNP cluster linked to a suspected vehicle where possible. The pale pink rows are clusters of human isolates only. Clusters 1–5 are colour coded to facilitate linking to the clusters highlighted in Figs 6 and 7. Clusters lacking a suspected vehicle or setting are designated with “NA” – not applicable.

Cluster	SNP level 5	Environmental	Food	Human	Other	Total no. of isolates	Suspected vehicle/setting
Cluster 1	1.1.1.3.300.639%	9	5			**14**	Sausage company
Cluster 2	1.1.12.21.21.22%	1	4		1	**6**	na
Cluster 3	1.1.307.463.559.606%	5		1		**6**	Butcher's shop outbreak
Cluster 4	1.1.17.33.445.482%		5			**5**	Unpasteurized cheese
Cluster 5	1.1.1.62.62.400%		4	1		**5**	Beef patty
Cluster 6	1.1.1.86.168.180%			4		**4**	na
Cluster 7	1.1.1.467.512.548%		2	2		**4**	Ice-cream food isolates
Cluster 8	1.1.308.470.516.552%	3				**3**	Catering – carvery
Cluster 9	1.1.1.443.466.502%	1	2			**3**	Butcher's shop outbreak
Cluster 10	1.1.1.38.38.41%			3		**3**	na
Cluster 11	1.1.8.114.552.597%		3			**3**	Sandwich manufacturer
Cluster 12	1.1.1.7.7.7%		1	1		**2**	Wholesaler meats
Cluster 13	1.1.17.33.489.524%		2			**2**	Dairy farm
Cluster 14	1.1.285.427.433.470%			2		**2**	na
Cluster 15	1.1.319.496.550.594%			2		**2**	na
Cluster 16	1.1.1.62.62.68%			2		**2**	na
Cluster 17	1.1.307.463.559.605%		2			**2**	Butcher's shop outbreak
Cluster 18	1.1.1.517.575.628%		2			**2**	Milk products – shop
Cluster 19	1.1.307.463.506.541%			2		**2**	Butcher's shop outbreak
Cluster 20	1.1.326.515.573.623%	1		1		**2**	na
Cluster 21	1.1.1.71.621.681%			2		**2**	na
Cluster 22	1.1.17.33.424.461%			2		**2**	na
Cluster 23	1.1.1.71.562.609%		2			**2**	Unpasteurized cheese – manufacturer

The majority of isolates exhibited a unique SNP type (*n*=218/237, 92%), and clusters of sequences at the 5 and 25 single linkage cluster level were sparsely populated. At the 5 SNP single linkage cluster level, there were 23 5 SNP single linkage clusters of at least two cases comprising 80/237 (34 %) isolates. Of these 23 clusters, 10/23 (43.5 %) included isolates sampled from food and/or environmental sources and no isolates from human cases, 8/23 (35 %) clusters comprised only human isolates, and 5/23 clusters contained isolates sampled from food and/or environmental sources and human cases (Table 2). The five largest clusters (*n*≥5 isolates) were designated clusters 1 to 5 (Table 2) and were coloured to match the corresponding cluster clades in Fig. 6. Fig. 6 highlights 20 of the phylogenetic clusters listed in Table 2, after excluding small environmental/food clusters (*n*=2 isolates – cluster 13, cluster 18 and cluster 23).

Expanding the 5 SNP single linkage cluster threshold to include isolates within the same 25 SNP single linkage cluster identified additional human cases linked with the suspected food and environmental source of contamination. One large cluster comprising entirely of human clinical isolates (*n*=19 cases) designated cluster 6 and the five clusters of human clinical isolates linked to a suspected vehicle (clusters 1–5 in Table 2) are described in detail below and highlighted in Fig. 7.

#### Epidemiological context of phylogenetic clusters of interest

Cluster 1 (red) was the largest 5 SNP single linkage cluster linked to a food source in this dataset with 14 isolates (Figs 6 and 7), comprising 5 isolates from food and 9 isolates from environmental sampling in January 2020 and early March 2020. These samples were linked to a single factory where meat products were processed and packed. When the cluster was expanded to the 25 SNP single linkage cluster level, six human isolates were incorporated in the cluster. Most of the human isolates were sampled from females (*n*=5/6), including three pregnancy-related listeriosis cases. The six human cases were notified prior to the sampling of the food and the factory. The cases were geographically and temporally dispersed, and did not fall within a 5 SNP cluster of each other or any food isolates at the time of notification; they were not investigated further. Following a retrospective analysis of the ESQs, none of the cases could be linked to a common food vehicle or other common exposure, and no confirmed links to the meat-processing factory were identified.

Cluster 2 (pink) contained four food isolates with sequences falling within a 5 SNP single linkage cluster, but temporally unrelated with respect to sample collection dates (Figs 6 and 7). No epidemiological data were available to assess geographical location or links to exposure. Expanding the investigation of the cluster to the 25 SNP single linkage cluster level did not reveal any further closely linked food, environmental or other clinical isolates.

Cluster 3 (orange) was a 5 SNP single linkage cluster comprising isolates from five environmental swabs taken from butcher's shop X, and one clinical case (female aged over 80 years) (Table 2, Figs 6 and 7). The environmental samples were collected between April and May 2019, and the human case was notified at the same time and lived within a 13 mile radius of butcher's shop X. Expanding this cluster to the 25 SNP threshold identified three additional human cases, two of which had confirmed consumption of food items from the implicated butcher's shop X.

Cluster 4 (purple) contained five isolates within the same 5 SNP single linkage cluster sampled from unpasteurized cheese made from cows' milk by cheese manufacturer X (Figs 6 and 7). Further analysis of the cluster at the 25 SNP single linkage cluster level revealed four isolates sampled from unpasteurized cows' milk products from two dairy farms in separate geographical regions of England and two additional clinical isolates. The two additional human isolates were not linked to unpasteurized dairy products at the time of notification, and we are unable to re-interview the cases retrospectively.

Cluster 5 (green) was a 5 SNP single linkage cluster of five isolates, one from a human clinical case and four isolates sampled from food. The food isolates were from beef patties from the same factory, collected in January 2016 and April 2017 (Figs 6 and 7). The human isolate originated from a female infant notified in September 2016 with no confirmed exposure to the food isolates. At the 25 SNP single linkage cluster threshold, an additional five human cases were included in cluster 5, notified between January 2016 and May 2020. Retrospective analysis of the food exposure history of the five additional cases did not reveal any links to beef patties.

Finally, cluster 6 (yellow) was the largest 5 SNP single linkage cluster composed of exclusively human isolates (Figs 6 and 7). No common exposures were identified. Expanding the cluster to include sequences clustering at the 25 SNP single linkage cluster level, identified 19 human isolates (female cases *n*=11/19, 58 %; male cases *n*=8/19, 42%) notified between March 2015 and June 2020. Of the 19 cases, 6/19 (32 %) were pregnancy related. In this cluster, three human cases were temporally linked in August 2017 but were geographically distant.

## Discussion

We reviewed the use of WGS data used for the surveillance of listeriosis in the UK at the UKHSA from January 2015 to December 2020. The isolates in the UKHSA archive belonged to a wide variety of CCs within either lineage I or lineage II. We observed a higher proportion *L. monocytogenes* isolated from human clinical cases in lineage I, and a higher proportion of food and/or environmental isolates in lineage II. This unequal distribution of isolates from human clinical cases across the population structure of *L. monocytogenes* and the clinical association with lineage I clones has been reported extensively worldwide, including studies in New Zealand, Chile and Ireland [20, 26–28]. Previous analysis of a global data set [20] found 51 % of the isolates in lineage I were from humans compared to only 19 % of the isolates in lineage II [20]. Conversely, the proportion of food isolates in lineage I was 37 % and in lineage II it was 69 % [20].

In this study, we focused on CC1 because it was the largest CC in lineage I and had the highest proportion of isolates linked to clinical cases. Previous studies have described the enhanced pathogenic potential of strains belonging to lineage I, and in particular those belonging to CC1, and the association of genomic traits facilitating invasive disease in humans, such as LIPI-1 and LIPI-3 [29, 39–41]. In this study, cases infected with CC1 were identified in elderly patients and individuals with pregnancy-related listeriosis. CC1 has been previously attributed to severe disease outcomes, including *L. monocytogenes*-associated respiratory infections in elderly immunocompromised patients [42], neurolisteriosis [43, 44], more severe inflammation and brain damage resulting from *Listeria* meningitis [45], sepsis, and miscarriage or stillbirth during pregnancy.

UKHSA collects surveillance data on clinical outcome for cases within England; however, it should be noted that for the most part clinical outcomes are self-reported by the patient or their family during follow up assessment. If patient death occurs after the assessment, the fatal outcome may not be captured. In addition, infection with *L. monocytogenes* is not always linked with cause of death. Patients with listeriosis can suffer from a multitude of severe complications capable of causing death, and it may be difficult to determine whether *L. monocytogenes* was the primary, contributory or an incidental to co-morbidity resulting in case fatality.

Surveillance reports on *L. monocytogenes* in the UK and elsewhere described increased listeriosis susceptibility in males, especially for infections of those over 75 years old [46–48]. Analysis by colleagues at the European Centre for Disease Prevention and Control (ECDC) (2021) reported a 60 % level of male infections [47]. Similarly, a study describing the patient demographics of listeriosis in the USA revealed a male incidence of 64 % [48]. This trend is consistent with the overall increased incidence rate reported by Public Health England in their 2018 and 2019 annual reports (57 %) [49, 50]. Our analysis revealed an overall higher proportion of female infections compared to males (59 % vs 41 %). The age/sex and clinical outcomes analysis in this study suggests the female bias of CC1 clinical cases was influenced by pregnancy-related listeriosis.

We found a higher proportion of notification of listeriosis in female infants compared to male infants. The Office for National Statistics reported the male-to-female birth ratio in the UK between 2015 and 2020 was 51 % male births and 49 % female births (https://www.ethnicity-facts-figures.service.gov.uk/uk-population-by-ethnicity/demographics/male-and-female-populations/). Therefore, the higher number of female neonatal listeriosis cases is noteworthy. Charlier *et al.* reported a higher proportion of male neonatal cases overall across all CCs of *L. monocytogenes* [46]; however, in our study, notification of listeriosis caused by CC1 were higher in female infants (75 % of neonatal cases). We also observed a high proportion of notifications were in females aged 20–39 years, and this correlates with the UK’s national average pregnancy age of 30–34 years old. Previous studies have reported an association with isolates belonging to CC1 and pregnancy-related listeriosis in humans and in ruminants [51, 52]. Charlier *et al.* report CC1 as the most abundant CC attributed to maternal and neonatal listeriosis [49, 53, 54].

Foodborne outbreaks of phylogenetically related isolates of *Escherichia coli* O157, *Salmonella* and *Campylobacter* species often exhibit clustering in time and may be geographically related [55, 56]. However, with respect to human infections of phylogenetically related isolates of *L. monocytogenes*, we rarely observed temporal (defined here as cases identified within 4 weeks of each other) or geographical (defined here as cases living withing 100 km radius of each other) clustering. This is most likely because the UKHSA's surveillance system only detects isolates from patients with severe clinical outcomes, due to underlying co-morbidities and/or a compromised immune system. Primary infection with *L. monocytogenes* in humans most commonly presents as an asymptomatic or mild infection, and only severe and invasive clinical infections of listeriosis are captured by the national surveillance systems. This results in a major bias in the population structure of the clones associated with human listeriosis.

Clusters of food and environmental isolates belonging to CC1 in the UKHSA database were sampled from ready-to-eat food items, dairy and processed meat products. Most recently in 2022, the latest L. monocytogenes outbreak isolated CC1 clones from a butcher’s shop in East Midland [57, 58]. Previously, Moura et al. described an association of CC1 clones with bovine meat and dairy products [25, 59]. Cantinelli *et al.*, in 2013, documented two outbreaks of CC1 in Switzerland and California between 1983 and 1987 caused by cheese made from cows' milk [60] and other studies have reported ruminants, especially cattle, being a common reservoir of hypervirulent CC1 clones [61, 62]. However, not all outbreaks of CC1 have been linked to ruminants; for example, an outbreak linked to contaminated coleslaw in Nova Scotia in 1981, and two outbreaks in France in 1992 and 2000 were caused by contaminated jellied pork tongue [60, 63].

Phylogenetic analysis of CC1 isolates in our collection highlighted the value of food and environmental sampling enabling us to link human cases to a contaminated food vehicle at the 5 SNP single linkage cluster level (for example, clusters 3 and 5). Expanding the cluster to include isolates falling within a 25 SNP single linkage cluster may provide a clue to a suspect food vehicle and/or route of transmission, not previously identified at the 5 SNP single linkage cluster level (for example, clusters 1 and 4). Due to *L. monocytogenes* ability to persist in an environment for months or even years [64], it is plausible that epidemiologically linked cases might be infected with isolates that are phylogenetically more distant from each other than we typically see for other foodborne pathogens. We showed that for CC1, increasing the genetic distance threshold linked in additional isolates from food and environmental sources and additional cases that, if linked to detailed epidemiological data, would provide clues to solving future outbreaks.

In this dataset, clusters comprising only human clinical cases were unresolved at the 5 SNP (clusters 2 and 6) and 25 SNP (cluster 6) single linkage cluster level. Despite reviewing the ESQs, which capture information of the clinical cases food history, clusters without isolates from food or from environmental sampling were challenging to resolve. This is due to the long incubation period of listeriosis. Symptoms can develop weeks after the exposure event, leading to poor patient recall; thus, confounding the capture of time-relevant food histories. Moreover, patients are often very unwell or deceased, and so interviewing the person or their bereaved family may not be possible. In addition, the clusters are sparse and notifications of cases within the same phylogenetic cluster are often months or even years apart, making it difficult to identify common food exposures. Even when a high-risk food item consumed by a recent case is identified, re-interviewing patients from historical cases to confirm the hypothesis is usually not an option.

Our analysis highlights the need to include isolates from human clinical infection, food and environmental sampling in the surveillance system. However, unlike human clinical cases, it is not mandatory to report food and environmental isolations of *L. monocytogenes*. Although local authorities will report positive isolations of *L. monocytogenes*, producers and food factories associated with private laboratories may not report their findings, unless connected with a specific survey. This results in a large gap in our dataset and hampers the public-health investigations and the prevention of ongoing infections of this clinically significant pathogen.

By analysing the population structure of *L. monocytogenes*, and integrating genomic and epidemiological data linked to cases, we showed that isolates belonging to CC1 caused the highest proportion of human clinical disease in the UK. We found that CC1 was commonly identified in females aged 20–39 with pregnancy-related listeriosis, and that the mortality rate increased in patients over the age of 60 in both sexes. Phylogenetic analysis revealed the population structure of CC1 in the UK comprised small, sparsely populated genomic clusters exhibiting little evidence of temporal or geographical clustering, except where isolates were linked to isolates from a specific food item and/or the same food-processing environments. In this CC1 dataset, only cases of infection with isolates that belonged to the same 5 or 25 SNP cluster as an isolate from the implicated food vehicle, or food-processing or farming environments, were resolved. This finding emphasizes the need for clinical, food and animal-health agencies to share sequencing data in real time and highlights the importance of a One Health approach to public-health surveillance.

## Supplementary Data

Supplementary material 1
