# Supplementary material for: Genomic epidemiology of the clinically dominant clonal complex 1 in the Listeria monocytogenes population in the UK
Source: Microb Genom. 2024 Jan 2;10(1):001155. doi: 10.1099/mgen.0.001155 (PMC10868620; doi:10.1099/mgen.0.001155)
Supplement: Supplementary material 1 [file mgen-10-1155-s001.pdf]

Strain_ID	Bioproject	Biosample	SRA accession
**293078**	PRJNA248549	SAMN09214416	SRR7172313
**165002**	PRJNA248549	SAMN32245354	SRR22746995
**891534**	PRJNA248549	SAMN14146609	SRR11123386
**293080**	PRJNA248549	SAMN32245363	SRR22746980
**891542**	PRJNA248549	SAMN14147081	SRR11123507
**129354**	PRJNA248549	SAMN32245343	SRR22747007
**293084**	PRJNA248549	SAMN09207764	SRR7163867
**892758**	PRJNA248549	SAMN14167289	SRR11148898
**129355**	PRJNA248549	SAMN32245342	SRR22746973
**127382**	PRJNA248549	SAMN32245344	SRR22747006
**892692**	PRJNA248549	SAMN14167247	SRR11148867
**127383**	PRJNA248549	SAMN32245348	SRR22747002
**177488**	PRJNA248549	SAMN32245352	SRR22746998
**131985**	PRJNA248549	SAMN32245349	SRR22747001
**894869**	PRJNA248549	SAMN14231283	SRR11195547
**308805**	PRJNA248549	SAMN32245357	SRR22746987
**893934**	PRJNA248549	SAMN14231832	SRR11195576
**893962**	PRJNA248549	SAMN14231281	SRR11195455
**893981**	PRJNA248549	SAMN32245356	SRR22746988
**150858**	PRJNA248549	SAMN32245375	SRR22746997
**198086**	PRJNA248549	SAMN32245340	SRR22746975
**893982**	PRJNA248549	SAMN14231704	SRR11195536
**406750**	PRJNA248549	SAMN09214590	SRR7172525
**893910**	PRJNA248549	SAMN14231459	SRR11195451
**856383**	PRJNA248549	SAMN13624877	SRR10729097
**293086**	PRJNA248549	SAMN09214415	SRR7172311
**198096**	PRJNA248549	SAMN32245351	SRR22746999
**198099**	PRJNA248549	SAMN32245347	SRR22747003
**201612**	PRJNA248549	SAMN32245368	SRR22746976
**201614**	PRJNA248549	SAMN32245367	SRR22746977
**201647**	PRJNA248549	SAMN32245346	SRR22747004
**214527**	PRJNA248549	SAMN32245365	SRR22746986
**214547**	PRJNA248549	SAMN32245350	SRR22747000
**205703**	PRJNA248549	SAMN32245341	SRR22746974
**208909**	PRJNA248549	SAMN32245345	SRR22747005
**246301**	PRJNA248549	SAMN10075732	SRR7850225
**208911**	PRJNA248549	SAMN32245366	SRR22746978
**214559**	PRJNA248549	SAMN32245373	SRR22747009
**214560**	PRJNA248549	SAMN32245374	SRR22747008
**232754**	PRJNA248549	SAMN09207807	SRR7164117
**238495**	PRJNA248549	SAMN10068787	SRR7842362
**241750**	PRJNA248549	SAMN10068755	SRR7842335
**248507**	PRJNA248549	SAMN09222152	SRR7179953

**250816**	PRJNA248549	SAMN09207759	SRR7163884
**293096**	PRJNA248549	SAMN09211254	SRR7167529
**265795**	PRJNA248549	SAMN09211282	SRR7167579
**265806**	PRJNA248549	SAMN09207779	SRR7163876
**278043**	PRJNA248549	SAMN10068670	SRR7842238
**279330**	PRJNA248549	SAMN09225585	SRR7184383
**279331**	PRJNA248549	SAMN09227774	SRR7187052
**282344**	PRJNA248549	SAMN32245362	SRR22746981
**282345**	PRJNA248549	SAMN32245361	SRR22746982
**289051**	PRJNA248549	SAMN09214491	SRR7172432
**293102**	PRJNA248549	SAMN09207762	SRR7163881
**293114**	PRJNA248549	SAMN09211261	SRR7167586
**295018**	PRJNA248549	SAMN09207770	SRR7163861
**298872**	PRJNA248549	SAMN10075690	SRR7850170
**304648**	PRJNA248549	SAMN09207760	SRR7163870
**308821**	PRJNA248549	SAMN09222267	SRR7180035
**308822**	PRJNA248549	SAMN09214506	SRR7172442
**306512**	PRJNA248549	SAMN09225620	SRR7184420
**306513**	PRJNA248549	SAMN10075764	SRR7850256
**308826**	PRJNA248549	SAMN10079757	SRR7866518
**308827**	PRJNA248549	SAMN10058186	SRR7827103
**306514**	PRJNA248549	SAMN10080075	SRR7866903
**308828**	PRJNA248549	SAMN10075846	SRR7850347
**308841**	PRJNA248549	SAMN09222428	SRR7180052
**308852**	PRJNA248549	SAMN09222218	SRR7180023
**315124**	PRJNA248549	SAMN09225626	SRR7184411
**318478**	PRJNA248549	SAMN09222250	SRR7180016
**329784**	PRJNA248549	SAMN09228600	SRR7187878
**329804**	PRJNA248549	SAMN10068853	SRR7842394
**329806**	PRJNA248549	SAMN10075837	SRR7850330
**329808**	PRJNA248549	SAMN10079753	SRR7866511
**329810**	PRJNA248549	SAMN10067050	SRR7841235
**330489**	PRJNA248549	SAMN32245358	SRR22746985
**335515**	PRJNA248549	SAMN32245371	SRR22746991
**340197**	PRJNA248549	SAMN09225678	SRR7184500
**340198**	PRJNA248549	SAMN09207799	SRR7164142
**342512**	PRJNA248549	SAMN10067159	SRR7841338
**351268**	PRJNA248549	SAMN10066929	SRR7841099
**351279**	PRJNA248549	SAMN09214414	SRR7172305
**351288**	PRJNA248549	SAMN10079791	SRR7866557
**351298**	PRJNA248549	SAMN10066377	SRR7839372
**351299**	PRJNA248549	SAMN10066976	SRR7841137
**359286**	PRJNA248549	SAMN09211269	SRR7167584
**361130**	PRJNA248549	SAMN10075795	SRR7850287

**363583**	PRJNA248549	SAMN09222180	SRR7179957
**365355**	PRJNA248549	SAMN09207785	SRR7164089
**367202**	PRJNA248549	SAMN09207783	SRR7164081
**368965**	PRJNA248549	SAMN09207769	SRR7163878
**367466**	PRJNA248549	SAMN10075680	SRR7850177
**371889**	PRJNA248549	SAMN09225624	SRR7184406
**376282**	PRJNA248549	SAMN09211259	SRR7167545
**379831**	PRJNA248549	SAMN32245353	SRR22746996
**387183**	PRJNA248549	SAMN09222208	SRR7180014
**388770**	PRJNA248549	SAMN09222246	SRR7180018
**389691**	PRJNA248549	SAMN10076040	SRR7850541
**392986**	PRJNA248549	SAMN09214651	SRR7172550
**396057**	PRJNA248549	SAMN09211255	SRR7167520
**397723**	PRJNA248549	SAMN09214644	SRR7172536
**399394**	PRJNA248549	SAMN09207792	SRR7164102
**401653**	PRJNA248549	SAMN09225584	SRR7184379
**406763**	PRJNA248549	SAMN10066981	SRR7841135
**406622**	PRJNA248549	SAMN09211271	SRR7167588
**408622**	PRJNA248549	SAMN09214698	SRR7172586
**409982**	PRJNA248549	SAMN09214672	SRR7172567
**411511**	PRJNA248549	SAMN09225741	SRR7184575
**412221**	PRJNA248549	SAMN09207828	SRR7164118
**411548**	PRJNA248549	SAMN09211344	SRR7167764
**411553**	PRJNA248549	SAMN10067766	SRR7841510
**418872**	PRJNA248549	SAMN09207836	SRR7164099
**413238**	PRJNA248549	SAMN09211310	SRR7167736
**417242**	PRJNA248549	SAMN10068817	SRR7842395
**417248**	PRJNA248549	SAMN10068779	SRR7842353
**422459**	PRJNA248549	SAMN09225685	SRR7184516
**428247**	PRJNA248549	SAMN09207831	SRR7164114
**436027**	PRJNA248549	SAMN09222211	SRR7180015
**444452**	PRJNA248549	SAMN09211301	SRR7167734
**444453**	PRJNA248549	SAMN09227821	SRR7187087
**444454**	PRJNA248549	SAMN09207833	SRR7164094
**445944**	PRJNA248549	SAMN09207794	SRR7164129
**455104**	PRJNA248549	SAMN09214502	SRR7172440
**455122**	PRJNA248549	SAMN10058224	SRR7827998
**455176**	PRJNA248549	SAMN10079837	SRR7866587
**460483**	PRJNA248549	SAMN09222141	SRR7179965
**461143**	PRJNA248549	SAMN10093714	SRR7879252
**460505**	PRJNA248549	SAMN09211262	SRR7167547
**476581**	PRJNA248549	SAMN09207806	SRR7164082
**482156**	PRJNA248549	SAMN09380432	SRR7277810
**488741**	PRJNA248549	SAMN09207830	SRR7164131

**488742**	PRJNA248549	SAMN09222252	SRR7180029
**501726**	PRJNA248549	SAMN09211309	SRR7167755
**505998**	PRJNA248549	SAMN09227820	SRR7187082
**557261**	PRJNA248549	SAMN09475598	SRR7416077
**556785**	PRJNA248549	SAMN09475601	SRR7416073
**578017**	PRJNA248549	SAMN32245359	SRR22746984
**580163**	PRJNA248549	SAMN10058243	SRR7827972
**581288**	PRJNA248549	SAMN10079713	SRR7866352
**585176**	PRJNA248549	SAMN10075689	SRR7850179
**582836**	PRJNA248549	SAMN10068650	SRR7842236
**585212**	PRJNA248549	SAMN10067030	SRR7841203
**585227**	PRJNA248549	SAMN32245355	SRR22746989
**587475**	PRJNA248549	SAMN10068682	SRR7842259
**587479**	PRJNA248549	SAMN10079797	SRR7866554
**591242**	PRJNA248549	SAMN10058184	SRR7827102
**609481**	PRJNA248549	SAMN10128134	SRR7902396
**616295**	PRJNA248549	SAMN10224419	SRR7989183
**620370**	PRJNA248549	SAMN11038554	SRR8647884
**624506**	PRJNA248549	SAMN11030927	SRR8645328
**627351**	PRJNA248549	SAMN11030863	SRR8645269
**636304**	PRJNA248549	SAMN11030985	SRR8645404
**654729**	PRJNA248549	SAMN32245372	SRR22746990
**661097**	PRJNA248549	SAMN11031004	SRR8645684
**661101**	PRJNA248549	SAMN11031136	SRR8645702
**668733**	PRJNA248549	SAMN11030992	SRR8645373
**678557**	PRJNA248549	SAMN11031025	SRR8645673
**688387**	PRJNA248549	SAMN11038572	SRR8647881
**692595**	PRJNA248549	SAMN11043576	SRR8657005
**697479**	PRJNA248549	SAMN11095645	SRR8704600
**704356**	PRJNA248549	SAMN20555908	SRR15340126
**707112**	PRJNA248549	SAMN11239034	SRR8771943
**730754**	PRJNA248549	SAMN11566974	SRR9004321
**735096**	PRJNA248549	SAMN11773515	SRR9088899
**735097**	PRJNA248549	SAMN11773507	SRR9088893
**735098**	PRJNA248549	SAMN11773511	SRR9088900
**735099**	PRJNA248549	SAMN11773508	SRR9088897
**735100**	PRJNA248549	SAMN11773524	SRR9088907
**735885**	PRJNA248549	SAMN11773510	SRR9088895
**740104**	PRJNA248549	SAMN11773525	SRR9088908
**741021**	PRJNA248549	SAMN11675339	SRR9083754
**741022**	PRJNA248549	SAMN11675337	SRR9083750
**752187**	PRJNA248549	SAMN11958693	SRR9201567
**755158**	PRJNA248549	SAMN12023329	SRR9273511
**756272**	PRJNA248549	SAMN12023326	SRR9273505

**758952**	PRJNA248549	SAMN12069715	SRR9306491
**758987**	PRJNA248549	SAMN12069728	SRR9306502
**761876**	PRJNA248549	SAMN12108587	SRR9335441
**769196**	PRJNA248549	SAMN12221012	SRR9647270
**772161**	PRJNA248549	SAMN12256447	SRR9672084
**781008**	PRJNA248549	SAMN12422898	SRR9886105
**781013**	PRJNA248549	SAMN12422925	SRR9886057
**786655**	PRJNA248549	SAMN12567590	SRR9961690
**789956**	PRJNA248549	SAMN12589688	SRR9989203
**788326**	PRJNA248549	SAMN12585032	SRR9973980
**802705**	PRJNA248549	SAMN32245376	SRR22746994
**809970**	PRJNA248549	SAMN12909917	SRR10228587
**816394**	PRJNA248549	SAMN12995146	SRR10247339
**816395**	PRJNA248549	SAMN12995155	SRR10247356
**885959**	PRJNA248549	SAMN14113740	SRR11091264
**839908**	PRJNA248549	SAMN13339010	SRR10501026
**839934**	PRJNA248549	SAMN13339008	SRR10501023
**844920**	PRJNA248549	SAMN13429415	SRR10559586
**845739**	PRJNA248549	SAMN13429400	SRR10559583
**848970**	PRJNA248549	SAMN13502221	SRR10600006
**867607**	PRJNA248549	SAMN13855187	SRR10886941
**869088**	PRJNA248549	SAMN13855184	SRR10886943
**875507**	PRJNA248549	SAMN13919344	SRR10962395
**875475**	PRJNA248549	SAMN13919375	SRR10962420
**883783**	PRJNA248549	SAMN14071225	SRR11048346
**883784**	PRJNA248549	SAMN14071226	SRR11048347
**888652**	PRJNA248549	SAMN14137882	SRR11113515
**888643**	PRJNA248549	SAMN32245364	SRR22746979
**888724**	PRJNA248549	SAMN14127726	SRR11102095
**888766**	PRJNA248549	SAMN14127727	SRR11102094
**888702**	PRJNA248549	SAMN14127720	SRR11102017
**888777**	PRJNA248549	SAMN14127714	SRR11102019
**888749**	PRJNA248549	SAMN14127691	SRR11102014
**920116**	PRJNA248549	SAMN14569674	SRR11514885
**912560**	PRJNA248549	SAMN14464436	SRR11430186
**912561**	PRJNA248549	SAMN14464434	SRR11430184
**912562**	PRJNA248549	SAMN14464433	SRR11430185
**912564**	PRJNA248549	SAMN14464432	SRR11430187
**912565**	PRJNA248549	SAMN14464427	SRR11430178
**912592**	PRJNA248549	SAMN14479756	SRR11442300
**912594**	PRJNA248549	SAMN14464431	SRR11430180
**943517**	PRJNA248549	SAMN14928007	SRR11791551
**945391**	PRJNA248549	SAMN14999202	SRR11832373
**952234**	PRJNA248549	SAMN15225473	SRR12005339

**957406**	PRJNA248549	SAMN15418777	SRR12128973
**960667**	PRJNA248549	SAMN15504734	SRR12195348
**962802**	PRJNA248549	SAMN15569707	SRR12251268
**963721**	PRJNA248549	SAMN15579361	SRR12264869
**973459**	PRJNA248549	SAMN15825365	SRR12457667
**988834**	PRJNA248549	SAMN16221591	SRR12676740
**988849**	PRJNA248549	SAMN16221593	SRR12676743
**995435**	PRJNA248549	SAMN32245360	SRR22746983
**998486**	PRJNA248549	SAMN16408266	SRR12803301
**1004102**	PRJNA248549	SAMN16451135	SRR12827959
**1019245**	PRJNA248549	SAMN16695351	SRR13004334
**1023032**	PRJNA248549	SAMN16785125	SRR13050047
**1030407**	PRJNA248549	SAMN16885771	SRR13128422
**1029800**	PRJNA248549	SAMN16849183	SRR13093696
**1032324**	PRJNA248549	SAMN16930264	SRR13153595
**1036563**	PRJNA248549	SAMN32245370	SRR22746992
**1036587**	PRJNA248549	SAMN32245369	SRR22746993
**1049283**	PRJNA248549	SAMN17120353	SRR13276790
